# Secreted protein acidic and rich in cysteine-like 1 suppresses metastasis in gastric stromal tumors

**DOI:** 10.1186/s12876-018-0833-8

**Published:** 2018-07-04

**Authors:** Chaoyong Shen, Yuan Yin, Huijiao Chen, Ruixue Wang, Xiaonan Yin, Zhaolun Cai, Bo Zhang, Zhixin Chen, Zongguang Zhou

**Affiliations:** 1 Department of Gastrointestinal Surgery, West China Hospital, Sichuan University, Chengdu, 610041 Sichuan China; 2 Department of Pathology, West China Hospital, Sichuan University, Chengdu, 610041 Sichuan China; 3 State Key Laboratory of Biotherapy and Cancer Center/Collaborative Innovation Center for Biotherapy, West China Hospital, Sichuan University, Chengdu, 610041 Sichuan China; 4 Institute of Digestive Surgery and State Key Laboratory of Biotherapy, West China Hospital, Sichuan University, Chengdu, 610041 Sichuan China

**Keywords:** Gastrointestinal stromal tumors, Microarray, SPARCL1, Malignization, Metastasis

## Abstract

**Background:**

Malignant growth and metastasis of gastrointestinal stromal tumors (GIST) occur in some patients even during the course of treatment, but their mechanisms remains poorly understand at the molecular level so far.

**Methods:**

Profiles of protein expression in gastric GIST tissues were explored using protein microarray analysis, down-regulation of SPARCL1 (secreted protein acidic and rich in cysteine-like protein 1) was validated by RT-qPCR, western blot and immunohistochemistry. The effect of specific shRNA-induced SPARCL1 downregulation on the biological traits of GIST 882 cell was investigated. We then employed a mouse xenograft model to investigate whether the low-expression of SPARCL1 impact the metastasis ability of GIST cells in vivo.

**Results:**

SPARCL1 was significantly downregulated in the gastric GIST with high-grade malignance as compared with low-grade malignance, its expression was closely correlated with tumor size, mitotic index, distant metastasis at the time of initial diagnosis and tumor progression of GIST (*P* < 0.05). Moreover, results of the Cox analysis showed that expression of SPARCL1 is an independent prognostic predictors for gastric GIST (*P* = 0.008; HR 0.157, 95% CI 0.040~ 0.612). Downregulation of SPARCL1 promoted cell migration and invasion, but did not affect proliferation, cell cycle and apoptosis of GIST 882 cells. In mouse xenograft model, GIST cells with the decreased expression of SPARCL1 presented an enhanced ability of liver metastasis (*P* < 0.05).

**Conclusions:**

Taken together, our present study demonstrated that SPARCL1 have a certain degree of malignancy-suppressing potential through inhibiting the metastasis of gastric GIST.

**Electronic supplementary material:**

The online version of this article (10.1186/s12876-018-0833-8) contains supplementary material, which is available to authorized users.

## Background

Gastrointestinal stromal tumors (GIST) is by far the most common mesenchymal neoplasm in the human digestive tract, which originating from the interstitial cells of Cajal or their progenitor cells [1, 2]. In the past few decades, despite tremendous efforts such as radical resection, targeted therapy and immunotherapy, have been made to improve the long-term outcome of GIST patients, the prognosis of advanced GIST patients is still unfavorable [3, 4]. The gain-of-function mutations of c-*KIT* or platelet-derived growth factor receptor alpha (*PDGFRα*) has been reported as the main cause of GIST instead of the primary promoter of the malignant potential of GIST [5]. Therefore, in addition to the *c-kit/PDGFRa* gene, other mechanisms must be involved in and ultimately determined the development and outcome of GIST.

Several risk-stratification schemes for defining malignant potential of GIST have been already proposed by researchers, which mainly comprise prognostic parameters such as tumor size, mitosis and tumor location, but these criteria are established merely rely on clinicopathological features and also do not clarify the biology mechanism underlying the clinical aggressiveness [6–9]. Undoubtedly, the mitotic rate and tumor size, which are powerful prognostic indicators for the risk assessment of GIST, are still key factors to distinguish different degrees of malignancy for those tumors with same locations. Additionally, as reported in previous study, gastric GIST have become malignant progression from preexisting less aggressive tumors, namely a stepwise progression from low- to high-grade malignancy [10]. The metastasis rate or tumor-related mortality for gastric GIST with tumor size ≤2 cm and mitotic rate ≤ 5 mitoses/50 HPFs and those with tumor size > 10 cm and mitotic rate > 5 mitoses/50 HPFs are 0.0 and 86.0%, respectively, according to the NCCN guidelines (Version 2. 2017). In other words, the former is more likely to be benign or low-grade malignancy while the latter is thought to be high-grade malignancy and may behave in an aggressive manner. However, the molecular events involved in GIST malignization remains unclear by far. As such, a better understanding of the molecular mechanism responsible for GIST metastasis is of critical significance, and would eventually result in new anticancer drug targets and greatly contribute to advances in diagnostic approaches.

To identify the candidate proteins which are closely related to the malignant biological potential of GIST, a common and straightforward microarray analysis was performed to clarify a list of differentially expressed proteins between low- and high-grade malignant gastric GISTs. Based on this approach, we found a potential novel candidate protein which was markedly down-regulated in high-grade malignant gastric GIST when compared to those with low-grade malignancy. We hypothesized that this protein might be mechanistically involved with the metastasis of GIST. Thus, we tested this idea for this candidate protein (secreted protein acidic and rich in cysteine-like protein 1, SPARCL1) and explored the relationship between SPARCL1 and gastric GIST progression. SPARCL1, which is also known as Hevin, MAST9, and SC1, is an extracellular matrix glycoprotein encoded by a conserved gene localized at chromosome 4q22 [11]. There is a wealth of evidences indicating that SPARCL1 participates in many physiological functions such as de-adhesive activity, cell proliferation, and facilitates lymphocyte transendothelial migration [12, 13]. SPARCL1 is expressed in a wide range of normal tissues and organs, such as lung, placenta, muscle, heart, lymphatic gland, colon, gastric mucosa and brain neurons. However, in contrast to its widespread expressed in normal tissues, downregulation of SPARCL1 has been reported as a putative tumor-suppressor factor in a wide variety of human malignancies including breast, colorectal, prostate and pancreatic cancers [13–19]. Furthermore, a few reports have shown that SPARCL1 inhibited prostate, colorectal and pancreatic cancer cell migration and invasion in vitro/vivo, suggesting that SPARCL1 may be a potential suppressor of metastatic progression in many cancers [13, 16, 17]. However, there is little known about the expressive characteristics of SPARCL1 as well as its potential role in the initiation and progression of GIST, particularly whether SPARCL1 can suppress the metastasis of GIST has not been addressed to date.

In this study, we aim to explore the expression pattern and clinicopathological significance of SPARCL1 in a Chinese gastric GIST cohort, as well as to investigate whether the downregulation of SPARCL1 can enhance the invasion/migration ability of GIST cells in vitro or facilitate liver metastasis of GIST cells in vivo.

## Methods

### Specimens and patients collection

To construct the protein expression profiles in gastric GIST, tumor and corresponding adjacent normal tissues were sampled from 4 primary gastric GIST patients (Table 1) during the surgical procedure. Moreover, additional 8 pairs of fresh gastric GIST and corresponding adjacent normal tissues were obtained from the Biological Specimen Banks (West China Hospital, Sichuan University, China.) to confirm the reliability of microarray results (Table 1). GIST tissues were categorized into low-grade malignancy (LGM, tumor size ≤2 cm and mitotic rate ≤ 5 mitoses/50 HPFs) and high-grade malignancy (HGM, tumor size > 10 cm and mitotic rate > 5 mitoses/50 HPFs), according to the NCCN guidelines. Additionally, formalin-fixed paraffin-embedded GIST specimens (*n* = 98) gathered from the Department of Pathology (West China Hospital, Sichuan University, from January 2010 to December 2013) were included in immunohistochemical analysis. In the present study, all samples were collected from patients without pre-treatment of radiotherapy and/or chemotherapy preoperatively as well as the history of other associated malignant tumors. The clinicopathological characteristics and follow-up data, including age at diagnosis, gender, hospital stay, tumor size, mitotic count, clinical symptom, tumor rupture, distant metastasis, risk classifications and so on, were collected from the patients’ medical records. Informed consents were provided by each patient before surgery, and the protocol of this study was approved by the Research Ethics Board of West China Hospital, Sichuan University, China.

**Table 1:** Clinicopathological characteristics of the 20 gastric GIST (No. 1~ 4 were used for microarray analysis and No. 5~ 20 were utilized to western blot/RT-qPCR analysis)

No.	Gender	Age (years)	Size (cm)	Mitoses (/50HPF)
1	Female	47	2 × 2	3
2	Male	62	2 × 1.8	2
3	Female	51	12 × 10	14
4	Male	35	15 × 12	16
5	Male	48	1.5 × 1	3
6	Male	35	2 × 1	2
7	Female	62	1.8 × 1.5	1
8	Male	52	2 × 1.5	2
9	Female	49	2 × 2	2
10	Female	58	1.5 × 1	3
11	Male	49	1 × 1	1
12	Female	61	2 × 1.8	2
13	Male	57	12 × 10	14
14	Female	46	15 × 12	16
15	Male	53	20 × 18	57
16	Male	51	15 × 13	12
17	Male	48	10 × 10	17
18	Male	63	11 × 10	> 20
19	Male	68	16 × 8	11
20	Female	42	15 × 10	18

### Protein microarray analysis

Human Cytokine Antibody Array (Raybiotech, Norcoss, GA, USA), which comprising 1000 cytokines, was utilized according to the manufacture’s instruction. Briefly, the arrays were blocked and incubated at room temperature for 30 min, incubated with biotin-conjugated antibodies for 1–2 h and with HRP-conjugated streptavidin for 2 h at room temperature. The membranes were incubated with chemiluminescent substrate and then exposed to x-ray film, signals were directly detected from membranes using chemiluminescene imaging system. The intensities of signals can be quantified by densitometry. Quantitative array analysis was performed using Array Vision Evaluation 8.0 (GE Healthcare Life Science, Little Chalfont, Buckinghamshire, UK). Proteins with significantly differential expression were selected with Fold change-value > 2.0 or < 0.5 and *P*-value < 0.05.

### Real-time quantitative PCR

Total RNA was extracted from the cultured cells and fresh-frozen gastric GIST tissues using the Trizol reagent (Invitrogen, CA, USA), according to the protocol of manufacturer. A NanoDrop ND-1000 spectrophotometer was utilized to determine the concentration and purity of isolated RNA. Real-time quantitative RCR reaction was performed using SYBR® Premix Ex Taq™ kit (Takara, Kyoto, Japan) as described by the manufacture. β-actin was used as an endogenous control. Primers were obtained from Invitrogen. The primers used for detection of SPARCL1 mRNA were 5′-ATG AAG CCA ACT CTG AAC ACG C-3′ (Forward) and 5′-ATG GTC CCC AGC CAA AAG C-3′ (Reverse), and for β-actin the primers were 5′-GTG GCC GAG GAC TTT GAT TG-3′ (Forward) and 5’-CCT GTA ACA ACG CAT CTC ATA TT-3′ (Forward). The PCR reactions were performed as follows: 95 °C for 10 min, then 40 cycles of 10 s at 95 °C and 30 s at 60 °C. All samples were run in triplicate, and the quantification of SPARCL1 was normalized to β-actin expression using the 2-ΔΔCt method.

### Protein extraction and western blot

Total cellular protein was extracted from fresh-frozen tumor tissues and cultured cells with a RIPA Lysis Buffer (Beyotime, Beijing, China) containing protease inhibitor, phenylmethylsulfonyl fluoride and phosphatase, protein concentration was measured using a BCA protein assay kit (Pierce, Rockford, IL, USA) in accordance with manufacturer’s instruction. Equal amounts of tissue or cell lysate per lane were loaded onto 20% sodium dodecyl sulfate polyacrylamide gels (SDS-PAGE). Proteins were then transferred to PVDF membranes (Millipore, Bedford, USA), and blocked with 5% skimmed milk under ambient temperature for 1 h. Membranes were incubated with rabbit polyclonal antibody specific for human SPARCL1 (1:1000 dilution, Abcam, Cambridge, United Kingdom) at 4 °C overnight, after washing three times with TBST, membranes were subsequently incubated with the horseradish peroxidase-conjugated goat anti-rabbit second antibody (Invitrogen) at a dilution of 1:5000 for 2 h at room temperature. Imaging was performed with a ChemiDoc™ XRS+ System (BIO-RAD, Hercules, CA, USA), and quantification was conducted using the pre-installed software. β-actin was used as loading control for normalization.

### Immunohistochemistry (IHC) and scoring of IHC

Briefly, paraffin-embedded samples were sliced into sections with thickness of 3–4 μm, which were then deparaffinized in xylene and rehydrated in gradually decreasing concentrations of alcohol to water. The tissue sections were heated in citrate buffer (pH 6.0) in a high-pressure cooker under the temperature of 95 °C for 5 min to retrieve antigen. Thereafter, the activity of endogenous peroxidase was blocked using 3% hydrogen peroxide, sections were incubated with primary rabbit polyclonal antibody against human SPARCL1 (1:100 dilution, Abcam) at 4 °C overnight, washed 3 times using phosphate-buffered saline (PBS) and then incubated with a secondary biotin-labeled antibody (ZSGB-BIO, Beijing, China) at 37 °C for 1.5 h. After washing again, sections were incubated with DAB-chromogen substrate mixture (DAKO, Glostrup, Denmark). Finally, sections were counterstained with hematoxylin, dehydrated in graded alcohol and xylene, mounted and coversliped. Immunostaining was evaluated individually and independently by two experienced pathologists using an Olympus CX31 microscope (Olympus, Tokyo, Japan) who were blinded to each other’s findings. The scoring was conducted according to ratio and intensity of positive-staining cells [20]. The mean percentage of positive tumor cells was assigned from 0 to 100% (< 10%, 0; 10~ 25%, 1; 26~ 50%, 2; 51~ 75%, 3; ≥76%, 4), staining intensity was scored as follows: (negative)% × 0 + (weak)% × 1 + (moderate)% × 2 + (intensive)% × 3. Finally, an IHC expressing score was generated for each case (staining ratio + intensity of staining). Low-expression of SPARCL1 was defined as score ≤ 3, whereas the slides with scored 4~ 7 were defined as SPARCL1 over-expression.

### GIST cell line and cell culture

Human GIST 882 line cells was obtained from Shanghai Cancer Institute (Shanghai, China). Cells were cultured in RPMI 1640 medium containing 20% fetal bovine serum (FBS) and 1% penicillin/streptomycin and incubated at 37 °C in a humidified atmosphere containing 95% air and 5% CO_2_.

### Construction of plasmids and cell transfection

GIST 882 cells were seeded into 6-well plates at a density of 5 × 10^5^ cells per well. SPARCL1-targeting double-stranded short hairpin RNA (shRNA) was cloned into the lentiviral vector (GV248, hU6-MCS-Ubiquitin-EGFP-IRES-puromycin). The scrambled sequences (shRNA 1: AGAGAAATAAAGTCAAGAA; shRNA 2: ACCCAATCTGATGATATTT; shRNA 3: ACCTATGCACCAGGTATTT) were synthesized by Genechem Corporation (Shanghai, China). All vectors were verified through sequencing. Lentiviral particles were produced as followings: transfected the lentiviral vectors into 293 T cells according to the manufacture’s instruction, collected supernatants after 48-h incubation, filtrated by 0.45 μm filters (Merck Millipore, Billerica, MA, USA), condensed by ultracentrifugation (Beckman Coulter TL-100, Miami, FL, USA). The harvested lentiviral particles were transfect GIST 882 cells based on manufacturer’s protocol using Lipofectamine 2000 (Invitrogen). The mRNA and protein levels of SPARCL1 in the transfected GIST 882 cells were evaluated by RT-qPCR and western blotting, respectively. Meanwhile, GIST 882 cells transfected with empty vectors in the same way were used as a negative control (Lv-shNC group). All transfecting experiments were conducted for three times. Subsequently, stable SPARCL1-knockdown GIST 882 cell line was established (Lv-shSPARCL1 group).

### Cell proliferation assay

After transfection, cells were harvested and reseeded in a 96-well plate under regular conditions with an initial density of 3 × 10^3^/well. A cell counting kit-8 cell proliferation assay (Dojindo, Kumamoto, Japan) was performed. The absorbing data at 450 nm were measured over next 3 days. All experiments were performed three times independently.

### Flow cytometry analysis of cell cycle and apoptosis

Flow cytometry was utilized to analyzed cell cycle and apoptosis. Cells were inoculated in 6-well culture plates at a density of 1 × 10^6^ cells per well. Following the pretreatments, the harvested cells were fixed with 70% ethanol at 4 °C overnight. Whereafter, cells were washed with PBS and then stained with propidium iodide (PI, 1 mg/mL). Flow cytometry (BD Biosciences, San Diego, CA, USA) was used to analyze the DNA content and comparing cells in G0/G1, S as well as G2/M stage.

For the additional analysis of cell apoptosis, an Annexin V-FITC apoptosis detection kit was used according to the manufacturer’s instruction. Cells were washed with ice-cold PBS, then 5 μL of PI (50 μg/mL), 5 μL of fluorescein isothiocyanate, and 100 μL of annexin V binding buffer were added to the cell suspensions and incubated in a dark condition at room temperature for 15 min. Flow cytometry was conducted to analyze the samples within 1 h. All assays were repeated in triplicate.

### In vitro cell migration

Wound-healing assay was adopted to evaluate the ability of cell migration, 3 × 10^5^ viable cells were seeded in 12-well plates and cultured with serum-free RPMI 1640 medium. After overnight incubation, the monolayer cells were scraped with a 1 mL sterilized pipette tip to form a linear wound. The plates were washed three times and cultured at 5% CO_2_ and 37 °C for another 24 h. Images were taken at the time of point of 0 h and 24 h, the wound area in each plate were detected using Image-Pro Plus 6.0 (IPP 6.0, produced by Media Cybernetics Corporation, USA) software. Wound-healing rate = [(scratch width at 0 h) - (scratch width at 24 h)]/(scratch width at 0 h)*100%.

### Transwell invasion assay

Assays were performed using 24-well plates with 8-μm polycarbonate filter membrane coated from bottom with Matrigel as an extracelluar matrix barrier. Cells (5 × 10^4^ cells per well) were seeded in the upper chambers and incubated with serum-free medium. Cell culture medium containing 20% FBS was added to the bottom of 24-well plates as a chemoattractant. After 24 h incubation at 37 °C, the non-migratory cells were removed from the upper surface by gentle scrubbing with a cotton tip. Subsequently, invading cells were fixed in 90% ethanol, stained with crystal violet and counted under a light microscopy. Each well counted 5 random fields, the experiments were carried out in triplicate.

### Construction of Xenografted nude mouse models

All animal experiments were conducted under an approved protocol from Sichuan University Institutional Animal Care and Use Committee. Experimental mice were housed and maintained in sterile environment. For subcutaneous Xenograft experiments, 5-week-old athymic nude mice (BALB/c^nu/nu^, *n* = 24) were randomized into three groups (half male and half female): normal control group, GIST Lv-shSPARCL1 group, and Lv-shNC group. Eight mice in each group were injected subcutaneously with 1 × 10^7^ cells into the costal region of naked mice. Animals were weighted at the initial of the experiment and the tumor size was measured every 3 days. Moreover, the volume of tumors (TV) was calculated as previously described [21]. Mice were sacrificed by cervical dislocation at day 32 postinjection to collect and weight the generated tumors. For hepatic metastasis mice model, thirty mice were kept at least 1 week before experiment manipulation and were grouped as mentioned above. After disinfection, mice were anesthetized with 1% pentobarbital sodium at a dosage of 75 mg/kg. The middle abdominal incision was carried out to expose the spleen. A total of 1 × 10^7^ viable tumor cells were implanted into splenic subcapsular space carefully to avoid extravasations, and gently pressed spleen injection point for minutes. Then, spleen was excised completely and abdominal incision was stitched. The mice were euthanized on day 30 postinjection or when the body weight was lost in more than 20% mice. Abdominal autopsy were performed to confirm hepatic metastatic nodules. The fresh tumor specimen and metastases were fixed with 10% formalin for the following pathologic examination.

### Statistical analysis and follow-up

All statistical analyses were determined using the Statistical Package for the Social Science (SPSS), version 21.0 for Windows (SPSS Inc., Chicago, IL, USA). Measurement data were expressed as mean ± standard deviation and enumeration data were described as percentage. Differences between groups were analyzed using analysis of variance for consecutive data and *χ*^2^ test or Fisher’s exact test for categorical variables. Cumulative survival was determined using the Kaplan-Meier method and log rank test. Progression-free survival (PFS) was defined from the start of any treatment until disease progression. Follow-ups were carried out by office visit, telephone call, or outpatient clinic visit from July 2016 to August 2016. The univariate and multivariate analyses were used to explore independent prognostic factors by Cox regression. Differences with two-sided *P* < 0.05 were considered as statistically significant.

## Results

### Microarray analysis of the gastric GIST patients with high- and low-grade malignancy

**Fig. 1:**
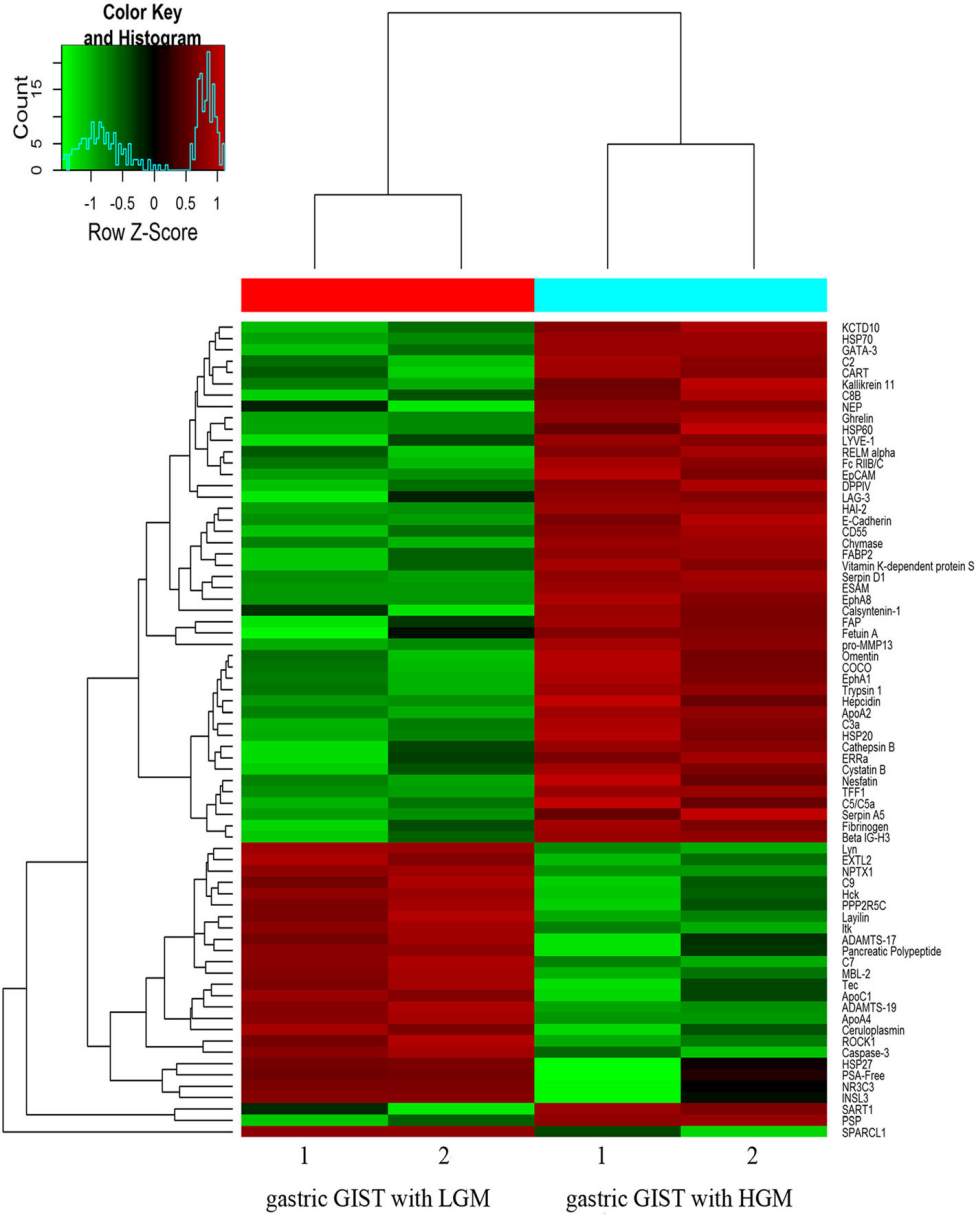
Heatmap of the hierarchical cluster analysis of the differentially expressed proteins between LGM and HGM in gastric GIST. Each column represents a single tissue specimen and each row represents a protein. Pseudocolors indicate differential expression, with red indicating high expression, green for low expression and black indicating for the mean expression levels

To investigate the underlying mechanisms for malignant transformation in GIST, protein expression profiles in four primary gastric GIST and corresponding adjacent normal tissues were analyzed using a protein microarray. After normalization, 64 out of 1000 proteins were significant differentially expressed in gastric GIST between low- and high-grade malignance. And 41 proteins were up-regulated, while 23 proteins were down-regulated in tumors with high-grade malignancy when compare with that of low-grade malignancy (fold change > 2 or < 0.5; *P* < 0.05). The top 10 up- and down-regulated proteins as compared to low-grade malignancy were shown in Tables 2 and 3, respectively. And then unsupervised hierarchical clustering classified them into two groups according to their protein expression (Fig. 1). The differentially expressed proteins were shown in the volcano plot (Additional file 1: Figure S1).

**Table 2:** Proteins significantly up-regulated in gastric GIST with HGM as compared to tumors with LGM

Name	Fold change	Standardized value in LGM	Standardized value in HGM	*P* value
pro-MMP13	15.746	8.000	125.969	0.012
FAP	8.385	11.359	95.250	0.036
FABP2	6.553	14.094	92.359	0.005
Chymase	5.991	16.953	101.563	0.001
PSP	5.564	3.656	20.344	0.005
Vitamin K-dependent protein S	5.338	14.250	76.063	0.023
Calsyntenin-1	4.666	12.875	60.078	0.031
HAI-2	4.643	18.531	86.047	0.000
Fetuin A	4.409	12.797	56.422	0.035
E-Cadherin	4.316	17.344	74.859	0.022

*GIST* gastrointestinal stromal tumors, *HGM* high-grade malignancy, *LGM* low-grade malignancy

**Table 3:** Proteins significantly down-regulated in gastric GIST with HGM as compared to tumors with LGM

Name	Fold change	Standardized value in LGM	Standardized value in HGM	*P* value
SPARCL1	25.099	134.906	5.375	0.002
INSL3	6.418	72.203	11.250	0.020
HSP27	6.051	216.141	35.719	0.031
NPTX1	5.722	118.469	20.703	0.003
PSA-Free	5.344	143.125	26.781	0.044
ROCK1	5.058	68.359	13.516	0.041
Ceruloplasmin	5.005	289.969	57.938	0.032
NR3C3	4.948	108.703	21.969	0.037
Hck	4.770	108.656	22.781	0.005
Lyn	4.165	117.016	28.094	0.001

*GIST* gastrointestinal stromal tumors, *HGM* high-grade malignancy, *LGM* low-grade malignancy

### Validation for differentially expression protein

**Fig. 2:**
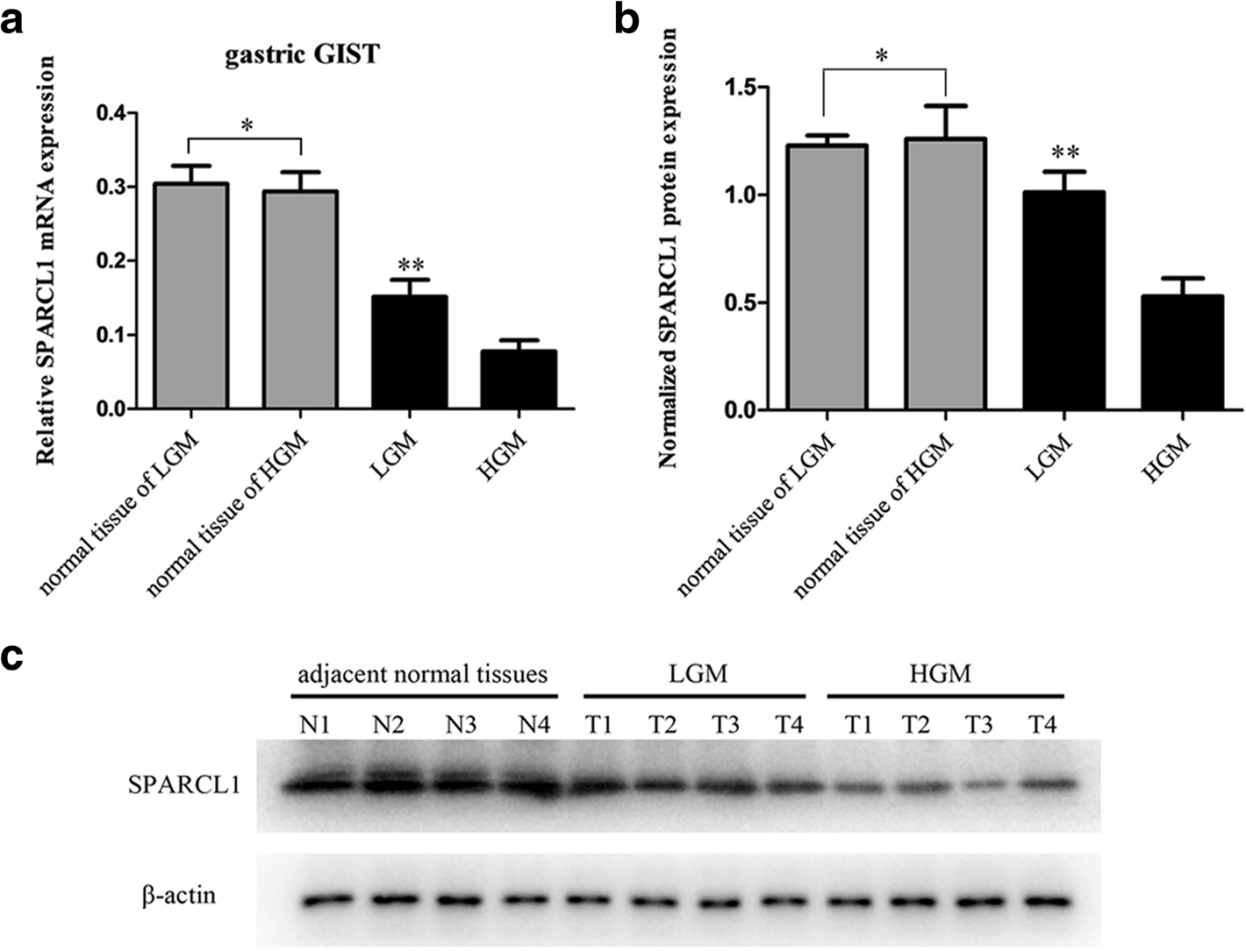
SPARCL1 is downregulated in human gastric GIST. The protein expression of SPARCL1 was determined by RT-qPCR and western blot with β-actin as a loading control. **a** Relative SPARCL1 mRNA level was significantly decreased in gastric GSIT with HGM as compared with those with LGM (**P < 0.05), while no statistical significance was noted between matched normal tissues from HGM and LGM (*P > 0.05); **b**, **c** A significant reduced expression of SPARCL1 in gastric GIST with HGM compared to those with LGM was determined by western blot analysis (**P < 0.05). N1~N2 for adjacent normal tissue of LGM, while N3~N4 for adjacent tissue of HGM. The bars represent mean ± SD (*n* = 4)

As mentioned above, SPARCL1 is expressed in many tissues and downregulated in a wide variety of human malignancies, which suggests that SPARCL1 might play a role as a tumor suppressor gene. Moreover, former microarray analysis showed that SPARCL1 was significantly downregulated in tumors with high-grade malignancy. Thus, to verify the microarray results and to evaluate the significance of the findings, SPARCL1 was chosen to examine the expression level by real-time quantitative PCR and Western blot in a larger cohort of 4 pairs of gastric GIST samples. Relative to a loading control β-actin, SPARCL1 mRNA levels were down-regulated in tumors of HGM in comparison to the samples with LGM (*P* < 0.05), which was in consistency with prior microarray data (Fig. 2a). In addition, high expression of SPARCL1 mRNA was observed in adjacent normal tissues in contrast to gastric GIST (*P* < 0.05). To determine whether SPARCL1 protein expression decreased in tumors of HGM, we also evaluated SPARCL1 expression by Western blot. SPARCL1 expression was decreased in tumors of HGM in accordance with microarray and PCR data (Fig. 2b, c). These data suggested that the downregulation of SPARCL1 in GIST might be responsible for the pathogenesis and progression, and then we further investigated the functional role of SPARCL1 in gastric GIST.

### Associations of SPARCL1 expression with Clinicopathological parameters in gastric GIST

**Fig. 3:**
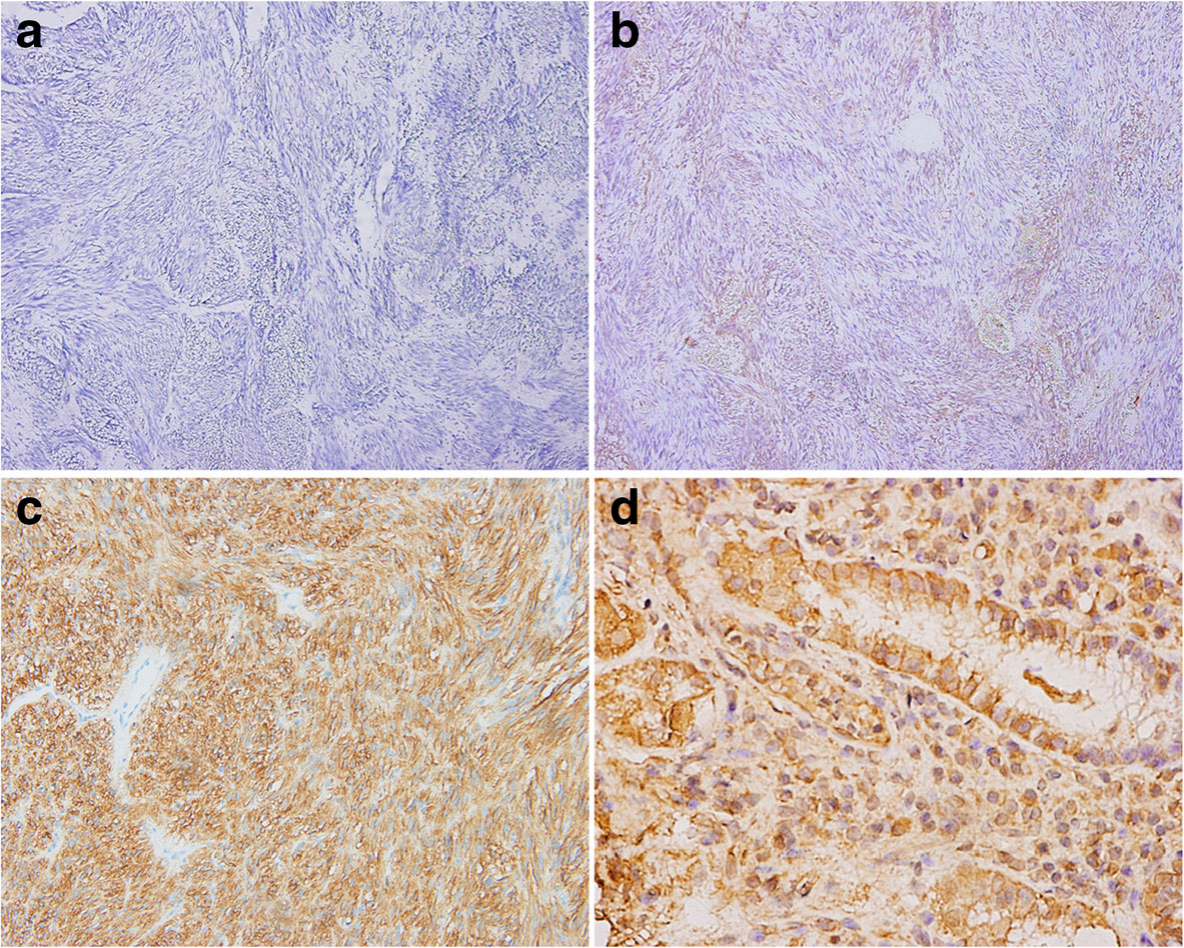
Representative immunohistochemical staining of SPARCL1 in gastric GIST and normal gastric tissues. **a**, **b** Negative/low expression of SPARCL1 in gastric GIST; (**c**, **d**) Gastric GIST and normal gastric tissues showed high expression of SPARCL1, separately. Original magnification: × 200 for **a**, **b**, **c** and × 600 for **d**

The entire cohort comprised 98 patients with gastric GIST and included 47 (47.96%) females and 51 (52.04%) males with a mean age of 57.28 ± 11.55 years and a median age of 58.8 years (range, 30~ 84 years). The mean tumor size was 7.37 ± 6.13 cm and with a median size of 6 cm (range, 1~ 35 cm). A total of 20 patients received adjuvant therapy with targeted drug (imatinib/sunitinib). Immunochemistry staining showed a significant SPARCL1 overexpression in normal gastric tissues, and SPARCL1 was mainly located in the cytoplasm. High expression of SPARCL1 was observed in a majority of cases (71/98), while lower expression of SPARCL1 was detected in 27 of 98 gastric GIST (Fig. 3). Table 4 indicates the association between SPARCL1 expression and clinicopathological characteristics in gastric GIST. Specifically, there was significantly correlation between SPARCL1 expression and tumor size, mitotic index (/50HPF), distant metastasis at the time of initial diagnosis (*P* < 0.05). In other words, the later clinical stage of tumor, the lower expressing of SPARCL1 would be. No significant differences, however, were noted between SPARCL1 expression and age, gender, and radical degree, etc.

**Table 4:** Association of SPARCL1 expression with clinicopathological factors of gastric GIST (*n* = 98)

Variables	SPARCL1 expression		*P* value
	SPARCL1-low	SPARCL1-high	
Gender			0.182
Male	10 (37.04)	37 (52.11)	
Female	17 (62.96)	34 (47.89)	
Age (years)	55.67 ± 14.11	57.89 ± 10.46	0.398
Surgical margins			0.062
R0	24 (88.89)	70 (98.59)	
Not R0	3 (11.11)	1 (1.41)	
Multivisceral resection (n, %)	7 (25.93)	2 (2.82)	0.002
Tumor rupture (n, %)	1 (3.70)	0 (0.00)	0.276
Hepatic metastasis at initial diagnosis (n, %)	5 (18.52)	0 (0.00)	0.001
Peritoneal metastasis at initial diagnosis (n, %)	7 (25.93)	0 (0.00)	< 0.001
Tumor size (cm)	11.94 ± 9.04	5.63 ± 3.24	< 0.001
Mitotic count (n, %)			< 0.001
≤5/50 HPF	3 (11.11)	35 (49.30)	
6~ 10/50 HPF	9 (33.33)	20 (28.17)	
> 10/50 HPF	15 (55.56)	16 (22.54)	
NIH risk classification (n, %)			0.127
Very low	0 (0.00)	4 (5.63)	
Low	1 (3.70)	20 (28.17)	
Intermediate	4 (14.81)	22 (30.99)	
High	22 (81.48)	25 (35.21)	

*SPARCL1* secreted protein acidic and rich in cysteine-like protein 1, *GIST* gastrointestinal stromal tumors, *HPF* high power field, *NIH* National Institutes of Health, “*Not R0*” includes surgeries of R1 and R2 resections

### Relationship between SPARCL1 expression and patient prognosis

**Fig. 4:**
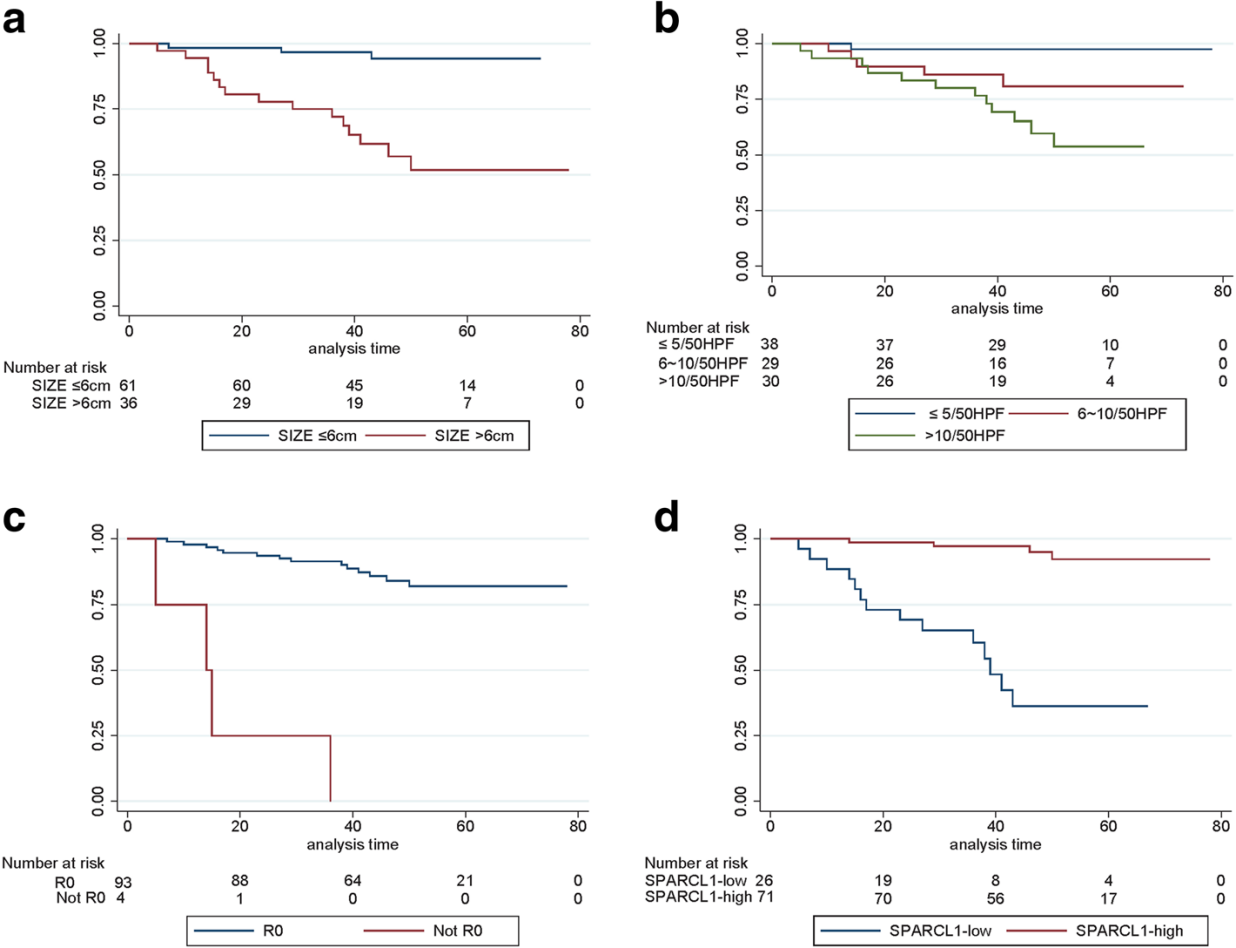
Kaplan-Meier survival curves of progression-free survival in patients with primary gastric GIST (*n* = 98). **a** Comparison of progression-free survival between tumors with ≤6 cm and > 6 cm (*P* < 0.001); **b** The tumors with mitotic count ≤5/50HPF showed significant better PFS compared with those of 6~ 10/50HPF and > 10/50HPF (*P* < 0.001); **c** Patients with R0 resection showed better PFS than that who did not achieve R0 resection (*P* < 0.001); **d** A worse prognosis was noted in patients with SPARCL-low expression in comparison to those with SPARCL1-high expression (*P* < 0.001)

A total of 6 patients died due to tumor progression or other causes for the entire cohort with a median follow-up of 49.5 months (range, 6~ 78 months). The 1, 2, 3 years progression-free survival rate of gastric GIST patients was 95.9, 89.8 and 86.5%, respectively. Factors associated with PFS by Kaplan-Meier univariate analysis were radical degree (*P* < 0.001), distant metastasis at the time of initial diagnosis (*P* < 0.05), tumor size (*P* < 0.001), mitotic count (*P* < 0.001), NIH risk classification (*P* < 0.001), and SPARCL1 expression (*P* < 0.001). We learned that low expression of SPARCL1 was related with poor prognosis for gastric GIST. The median survival was not achieved for patients with SPARCL1-high expression versus 36 months (range, 0~ 67 months) for patients with SPARCL1-low expression (*P* < 0.001, Fig. 4). By incorporating these factors, except the highly collinear variables, into the Cox multivariate regression proportional hazards model, we found that radical degree (HR 7.266, 95% CI 1.222~ 43.221; *P* = 0.029), tumor size (HR 4.518, 95% CI 1.172~ 17.410; *P* = 0.028), and SPARCL1 expression (HR 0.157, 95% CI 0.040~ 0.612; *P* = 0.008) were independent prognostic predictors in gastric GIST (Table 5). Above findings suggested that SPARCL1 may function as a tumor suppressor in gastric GIST.

**Table 5:** Univariate and multivariate analysis of factors with PFS in gastric GIST using Cox proportional hazards regression modeling (*n* = 98)

Variables	*P* univariate	Multivariate analysis	
		HR (95% CI)	*P*
Surgical margins			
R0		Reference	
Not R0	< 0.001	7.266(1.222~ 43.221)	0.029
Multivisceral resection			
No		Reference	
Yes	< 0.001	1.553(0.439~ 5.489)	0.495
Peritoneal metastasis at initial diagnosis			
No		Reference	
Yes	< 0.001	2.201(0.512~ 9.466)	0.289
Hepatic metastasis at initial diagnosis			
No		Reference	
Yes	0.003	2.281(0.384~ 13.533)	0.364
Tumor size			
≤6 cm		Reference	
> 6 cm	< 0.001	4.518(1.172~ 17.410)	0.028
NIH risk classification			
Very low/low		Reference	
Intermediate/high	0.005	1.681(0.309~ 9.155)	0.548
Mitotic count			
≤5/50 HPF		Reference	
6~ 10/50 HPF		3.752(0.379~ 37.105)	0.258
> 10/50 HPF	< 0.001	8.924(0.908~ 87.698)	0.060
SPARCL1 expression			
Low		Reference	
High	< 0.001	0.157(0.040~ 0.612)	0.008

*PFS* progression-free survival, *GIST* gastrointestinal stromal tumors, *HR* hazard ratio, *CI* confidential interval, *HPF* high power field, *NIH* National Institutes of Health, *SPARCL1* secreted protein acidic and rich in cysteine-like protein 1, “*Not R0*” includes surgeries of R1 and R2 resections

### Transfecting cells with shRNA for knocking down SPARCL1

**Fig. 5:**
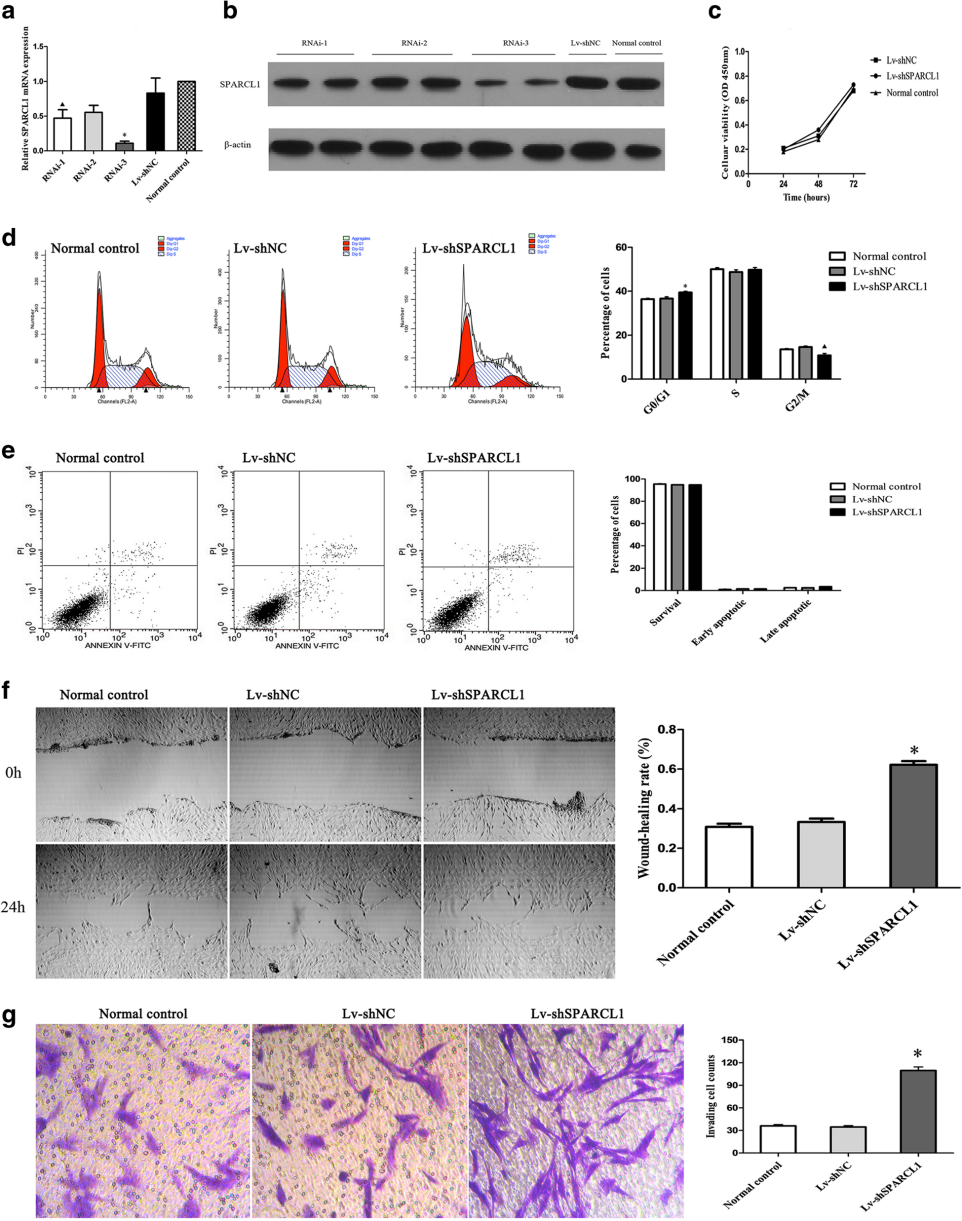
The roles of SPARCL1 knockdown in GIST 882 cell proliferation, cell cycle, apoptosis, migration and invasion. **a**, **b** Downregulation of SPARCL1 in GIST cells was confirmed by RT-qPCR and western blot. ▲compared with RNAi-3, *P* < 0.05; * compared with RNAi-2, *P* < 0.05. **c**: A CCK-8 assay was performed to evaluate the influence of SPARCL1 knockdown on GIST 882 cell proliferation. **d**: SPARCL1 downregulation could induce the arrest of cell cycles in G0/G1 phase. ▲ * compared with the other two groups, both *P* < 0.05. **e** SPARCL1 downregulation did not affect apoptosis of GIST 882 cell. **f** Wound-healing assay showed that SPARCL1 knockdown promoted migration of GIST 882 cells after cultured for 24 h. * compared with the other two groups, both *P* < 0.05. **g** The invasive GIST cells and quantitation of invasive GIST cell counts. * compared with the other two groups, both *P* < 0.05. Data represent the mean of three independent experiments, and the error bars refer to SD from the mean

The moderate abundance of SPARCL1 expression was detected in cultured GIST 882 cells (parental cell) by qPCR. So, to investigate the functional significance of SPARCL1 knockdown on GIST 882 cells, a lentivirus shRNA-based system was used, which resulting in Lv-shSPARCL1 and Lv-shNC. The relative transcribing and expressing quantities of SPARCL1 in the group treated with shRNA were significantly lower to those in the normal control (*P* < 0.05) and Lv-shNC group (*P* < 0.05) after normalizing to β-actin (Fig. 5a, b). Because the interference efficiency of RNAi-3 was more efficient than the others, so a series of in vitro functional assays were performed by using this interference group.

### Effects of SPARCL1 downregulation on GIST cell proliferation, cell cycle and apoptosis

CCK-8 cell proliferation assay was conducted to determine the changes of SPARCL1 knockdown on GIST cell growth. According to the Fig. 5, the proliferation rates of the Lv-shSPARCL1, Lv-shNC and normal control group were similar in 24-, 48- and 72-h measurements (*P* > 0.05), which demonstrating that downregulation of SPARCL1 had no significant effect on GIST 882 cell growth in vitro. The influence of SPARCL1 silencing on cell-cycle progression was determined by flow cytometry with the aim of observing the changes in cell cycles induced by knocking out SPARCL1. The ratio value of cells in the G0/G1 phase in the Lv-shSPARCL1 group was significantly higher than those in the Lv-shNC and normal control group (*P* < 0.05), while the percentages of cells in the G2/M phase decreased in the Lv-shSPARCL1 group, and which indicated that SPARCL1 silencing could induce the arresting of cell cycles in G0/G1 phase. Additionally, SPARCL1 downregulation did not affect apoptosis of GIST 882 cell among three groups (Lv-shSPARCL1, Lv-shNC and normal control group, *P* > 0.05).

### Downregulation of SPARCL1 suppresses migration and invasion of GIST 882 cells

The ability to migrate or invade was considered as the main malignant phenotype and prerequisite for tumor cell metastasis. To determine whether downregulation of SPARCL1 changed cell motility, an in vitro wound healing assay was utilized. In wound healing assay, it was shown that the cell migration abilities of GIST 882 was remarkably promoted after knocking out of SPARCL1 in comparison with those in the normal control and Lv-shNC group at 24 h (*P* < 0.001). Moreover, we also investigated the SPARCL1 effect on invasive capability of GIST 882 cells. In a 24-h transwell cell invasion assay, there were more Lv-shSPARCL1 cells passing through membranes coated with Matrigel compared to Lv-shNC and normal control group (*P* < 0.001, Fig. 5). The SPARCL1 notably decreased the invasion ability of GIST 882 cells by 3.2-fold when compared with vector control cells (*P* < 0.001), while no statistical significance was observed between the Lv-shNC and normal control group (*P* > 0.05). Collectively, these data support a role for SPARCL1 in reducing abilities of migration and invasion in GIST 882 cell line.

### Effect of SPARCL1 on GIST growth and liver metastasis in a mouse xenograft model

**Fig. 6:**
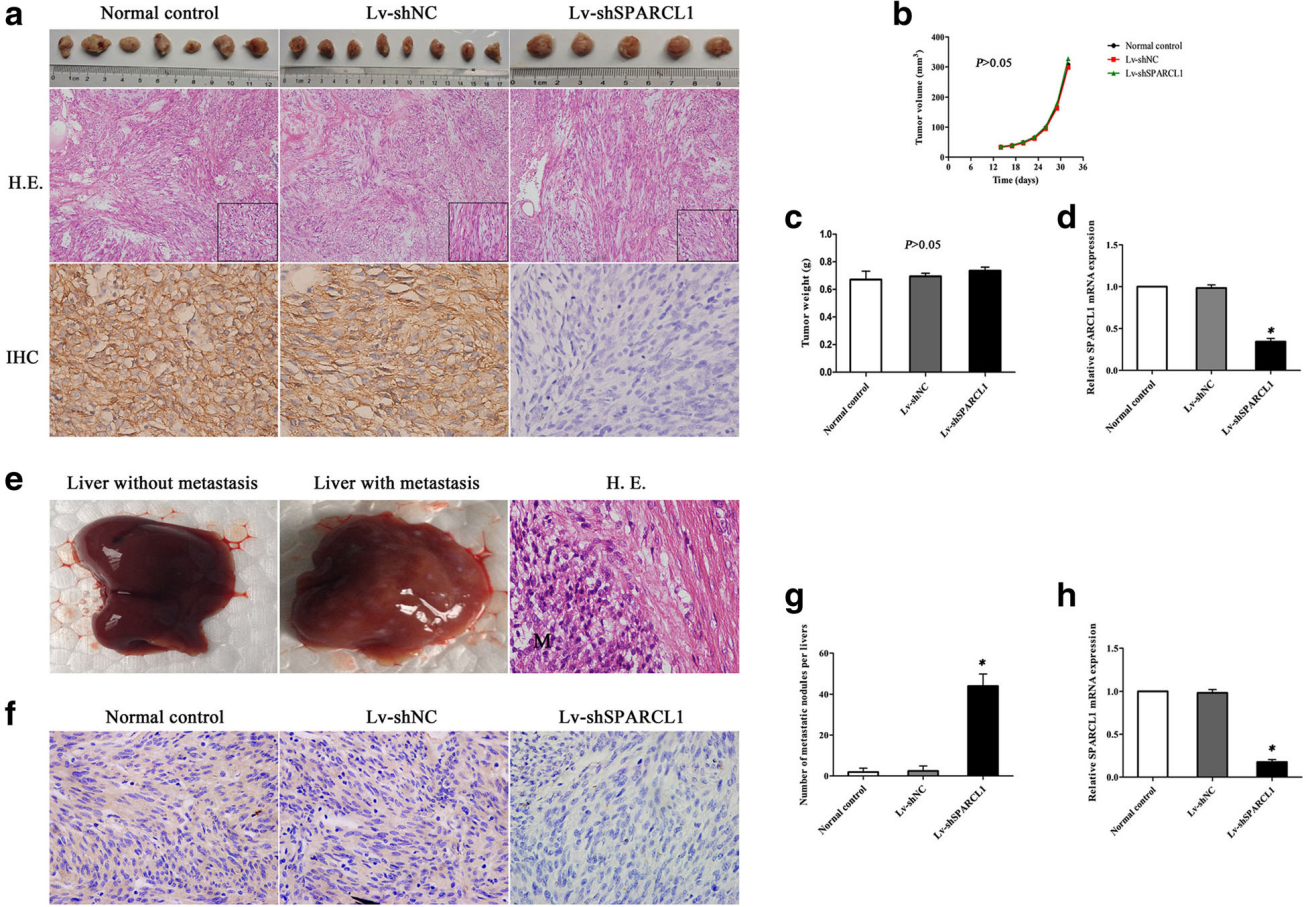
Downregulation of SPARCL1 in GIST 882 cells promoted liver metastasis in xenograft mouse models. **a** Representative images of hematoxylin and eosin staining (HE × 100) and IHC staining (× 600) of SPARCL1 in xenografted tumors; **b**, **c** Quantification of tumor volume and tumor weight for tumorigenesis among three groups in nude mice (*P* > 0.05). The error bars refer to SD (*n* = 7, 8, and 5 for normal control, Lv-shNC and Lv-shSPARCL1, respectively); **d** Expression level of SPARCL1 mRNA was significantly decreased in Lv-shSPARCL1 group as compared to other two groups (*P < 0.05). The error bars present SD (*n* = 7, 8, and 5 for normal control, Lv-shNC and Lv-shSPARCL1, respectively); **e** Representative of gross view of liver without and with metastasis, and HE staining of liver metastatic tumor (M); **f** Low expression of SPARCL1 in liver metastatic nodules was observed in Lv-shSPARCL1 group as compared to other two groups; **g** The number of liver metastatic nodules was increased in Lv-shSPARCL1 group when compared to other two groups (*P < 0.05); **h** The relative SPARCL1 mRNA level of liver metastatic nodules of nude mice (*P < 0.05). The error bars refer to SD from the mean

To gain insight into whether SPARCL1 impacts tumor growth and metastasis in vivo, mouse xenograft models were performed by means of implanting Lv-shSPARCL1, Lv-shNC and parental cells. The subcutaneous model in nude nice was successfully established, and the tumor volume was monitored every 3 days from 14 days after the injection of these cells. No tumorigenesis were observed in 1 and 3 nude mice for the normal control group and Lv-shSPARCL1 group, respectively. There was no statistical significance in terms of average tumor volume and tumor weight among three groups (*P* > 0.05). The SPARCL1 expression for xenograft groups was verified by RT-qPCR and IHC staining, and it was found that SPARCL1 was downregulated in the Lv-shSPARCL1 xenograft group as compared with that of the Lv-shNC and normal control group. Moreover, the representative images for the hematoxylin and eosin staining were shown in Fig. 6. Due to the liver accounts for the most of the GIST metastases, the intrasplenic implantation was conducted to assess the influence of SPARCL1 on hepatic metastasis. There were 1, 1 and 6 nude mice occurred liver metastasis in the normal control group, Lv-shNC group and Lv-shSPARCL1 group, respectively. Review of metastasis from sacrificed mice indicated that the metastatic nodules formation was significantly higher in the Lv-shSPARCL1 group than those in the Lv-shNC and normal control group (*P* < 0.05). Furthermore, the expression of SPARCL1 in each tissue sample was determined by RT-qPCR and IHC staining (Fig. 6). These results demonstrated that SPARCL1 downregulation could inhibit the development of liver metastasis of GIST cells in vivo.

## Discussion

SPARCL1 is a secreted matricellular glycoprotein belonging to SPARC family, which plays important roles in the regulation of cell adhesion, proliferation, cell cycle, and migration [13, 22]. In recent years, a multitude of studies have reported that SPARCL1 implicated in the initiation and progression of various cancer, including prostate, colorectal, gastric, liver, breast cancer [23, 24]. Here, we first explored the molecular basis of GIST malignization by studying the expression profile of proteins, and found that SPARCL1 was expressed at lower level in high-grade malignance gastric GIST compared with low-grade ones. The Kaplan-Meier and Cox proportional hazard analyses demonstrated that SPARCL1 can be considered as a protective factor in gastric GIST. In vitro and in vivo experiments suggested that downregulation of SPARCL1 significantly enhanced the invasion and liver metastasis ability of GIST 882 cell, but did not influence the GIST cell proliferation, cell cycle, and apoptosis. These studies validate that SPARCL1 may play a crucial role in gastric GIST progression.

According to the NCCN guidelines (Version 2. 2017), all GIST have potential malignancy-transforming ability even if the tumor is small and asymptomatic [25]. Several risk-stratification systems were proposed, which incorporated the prognostic factors such as tumor location, mitotic count and tumor size, to assess the malignant potential or risk level of GIST in clinics. However, the intrinsic mechanism in progression of GIST is not well defined to date. To this end, in the present work, we explored differential expression of proteins which might be involved in gastric GIST malignization, and further validation of selected candidate (SPARCL1) was performed. Identifying molecular biomarkers associated with the recurrence risk of GIST could contribute to novel treatment approaches, and also complement the existing risk evaluation criteria [26].

Growing evidences show that SPARCL1 often presents a reduced or absent expression pattern in many human epithelial cancers, suggesting its role as tumor suppressor [16, 17]. In 2012, Li and colleagues reported that both SPARCL1 mRNA and protein levels were relevantly downregulated compared to normal tissues in a large series of patients harboring gastric cancer [27]. In agreement with their findings, data from both PCR and western blot analyses indicated that SPARCL1 was decreased in gastric GIST compared with normal gastric tissues; and its expression level was decreased with the increase of malignancy in gastric GIST. However, an increased expression of SPARCL1 was noted in a few of other types of human tumors which derived from liver, colon and rectum when compared to corresponding healthy tissues [15, 23]. These reports indicating that SPARCL1 exactly participates in cancer occurrence and development, but its expression pattern as well as functionary mechanisms in various tumor conditions may be different.

Pilot studies have analyzed the associations of SPARCL1 expression with the clinicopathological factors. As reported, Loss of SPARCL1 was more observed in N1/2-stage and high/moderate-differentiated tumors than in N0-stage and poor/un-differentiated hilar cholangiocarcinoma, respectively [28]. Additionally, expression of SPARCL1 was closely associated with tumor grade, tumor size, regional lymph nodes, and TNM stages in gastric cancer [27]. In the present study, an analysis of the clinicopathological features showed that low expression ratio of SPARCL1 markedly increased tumor size, mitotic index (/50HPF), and number of distant metastasis at the time of initial diagnosis and tumor progression. The aforementioned parameters are known to be factors for evaluating recurrence risk or malignant potential of GIST. Thus, our study indicates that SPARCL1 expression is negatively related to the malignant biological behavior in gastric GIST. Moreover, the prognostic value of SPARCL1 has been extensively investigated in multiple solid tumors [16, 17, 19, 27, 28]. High expression of SPARCL1 in prostate cancer is associated with better survival, 5 year metastatic disease-free survival of men with loss of SPARCL1 expression had 5 year metastatic disease-free survival of ~ 60% vs. ~ 80% for men with high SPARCL1 expression [16]. A Swedish research team reported that over-expression of SPARCL1 protein in the primary colorectal carcinomas had a short survival [15], while the diametrically opposing conclusions were drawn by Hu and colleagues through western blot and immunohistochemistry [17]. Thus, its specific role in GIST still awaits further confirmation. In our study, the Kaplan-Meier analysis revealed that low expression of SPARCL1 was related with poor prognosis for gastric GIST, which was consistent with previous reports. The median survival was not achieved for patients with SPARCL1-high expression versus 36 months (range, 0~ 67 months) for patients with SPARCL1-low expression. Furthermore, in addition to the clinicopathological factors, we have introduced biomarker, SPARCL1, into the Cox regression model, and found that SPARCL1 expression functions as a protective factor (HR 0.157; 95%CI 0.040~ 0.612; *P* = 0.008).

Metastasis is the main cause of cancer-related death. The hallmarks of cancer comprise six biological capabilities: sustaining proliferative signaling, evading growth suppressors, evasion of apoptosis, limitless replicative potential, inducing angiogenesis, and ability to invade and metastasize [29]. It is worthy that the final hallmark of caner, invasion and metastasis, underlies its deadly progressive nature. Many tumors prefer certain organs, particularly the lungs, bone marrow, and liver, as metastatic sites. Specifically, cancerous cells preferentially metastasized to liver tissue, which caused the high recurrence (about 55~ 72%) and low survival rates to patients with GIST [30, 31]. Given the above findings, which showed the malignancy-suppressing ability of SPARCL1, we further examined whether downregulation of SPARCL1 affects GIST cell invasion and migration in vitro and in vivo. Our results suggested that SPARCL1 suppressed the invasion and migration of GIST cell in vitro, but did not inhibit cell proliferation and apoptosis. Results from in vivo study showed that downregulation of SPARCL1 significantly promoted the ability of liver metastasis, suggesting that SPARCL1 may participate in the progression of gastric GIST, which was consistent with previous reports [14, 16, 17]. Hurly et al. demonstrated that SPARCL1 blocks the activation of the Ras homolog gene family, member C, thereby inhibiting cellular movement [16]. In addition, SPARCL1 may also promote differentiation possibly via mesenchymal-epithelial transition, which inhibits the aggressiveness of colorectal cancer [17]. Besides, unlike the influence to colorectal and pancreatic cancer cells, SPARCL1 did not inhibit the proliferation of hilar cholangiocarcinoma cells and prostate cells in vitro [13, 14, 16, 17, 28]. Our study demonstrated a crucial role for SPARCL1 in facilitating GIST invasion and migration both in vitro and vivo, strongly suggested SPARCL1 functioning as a metastatic suppressor and may serve as a potential therapeutic target for patients with metastatic gastric GIST. However, the detail mechanism of SPARCL1 involving in GIST malignization should be explored in further study, which is also the major limitation of this study.

Downregulation of SPARCL1 in human tumors has not been well addressed yet. The possible mechanism of gene inactivation may result from gene mutations, loss of heterozygosity (LOH), and epigenetic alteration such as promoter methylation. Esposito and colleagues found that promoter demethylation slightly increased SPARCL1 mRNA, suggesting that hypermethylation is not the key mechanism accounting for low expression of SPARCL1 in pancreatic cancer cell [13]. Meanwhile, result from a previous study showed that no mutation or deletion that might be responsible for downregulation of SPARCL1 was noted in lung tumor [32]. In a recent study, Li et al. showed that no methylation variable positions and no mutation were observed in gastric cancer, but a possible mechanism involving the LOH of SPARCL1 gene was revealed [27].

## Conclusions

Overall, the collective findings from our study show for the first time that SPARCL1 might have a certain degree of malignancy-suppressing potential, which suppresses metastasis of gastric GIST. Additionally, this study also sets the stage for further investigations on the basic mechanisms that underlie GIST metastasis.

## Additional file

Additional file 1: **Figure S1.** Volcano plots of differential expression proteins. The vertical lines correspond to 2.0-fold up and down, respectively, and the horizontal line represents a *p*-value of 0.05. The red point in the plot represents the differentially protein with statistically significance. (A: gastric GIST with LGM; B: gastric GIST with HGM; C: corresponding adjacent normal tissues for LGM; D: corresponding adjacent normal tissues for HGM). (JPG 751 kb)

## Abbreviations

GIST: Gastrointestinal stromal tumors
HGM: High-grade malignancy
HPFs: High power field
HR: Hazard ratio
IHC: Immunohistochemistry
LGM: Low-grade malignancy
NCCN: National Comprehensive Cancer Network
PCR: Polymerase chain reaction
PDGFRa: Platelet-derived growth factor receptor alpha
PFS: Progression-free survival
SPARCL1: Secreted protein acidic and rich in cysteine-like protein 1
TV: Volume of tumor
