# Supplementary figures and images for: Secreted protein acidic and rich in cysteine-like 1 suppresses metastasis in gastric stromal tumors
Source: BMC Gastroenterol. 2018 Jul 4;18:105. doi: 10.1186/s12876-018-0833-8 (PMC6030747; doi:10.1186/s12876-018-0833-8)

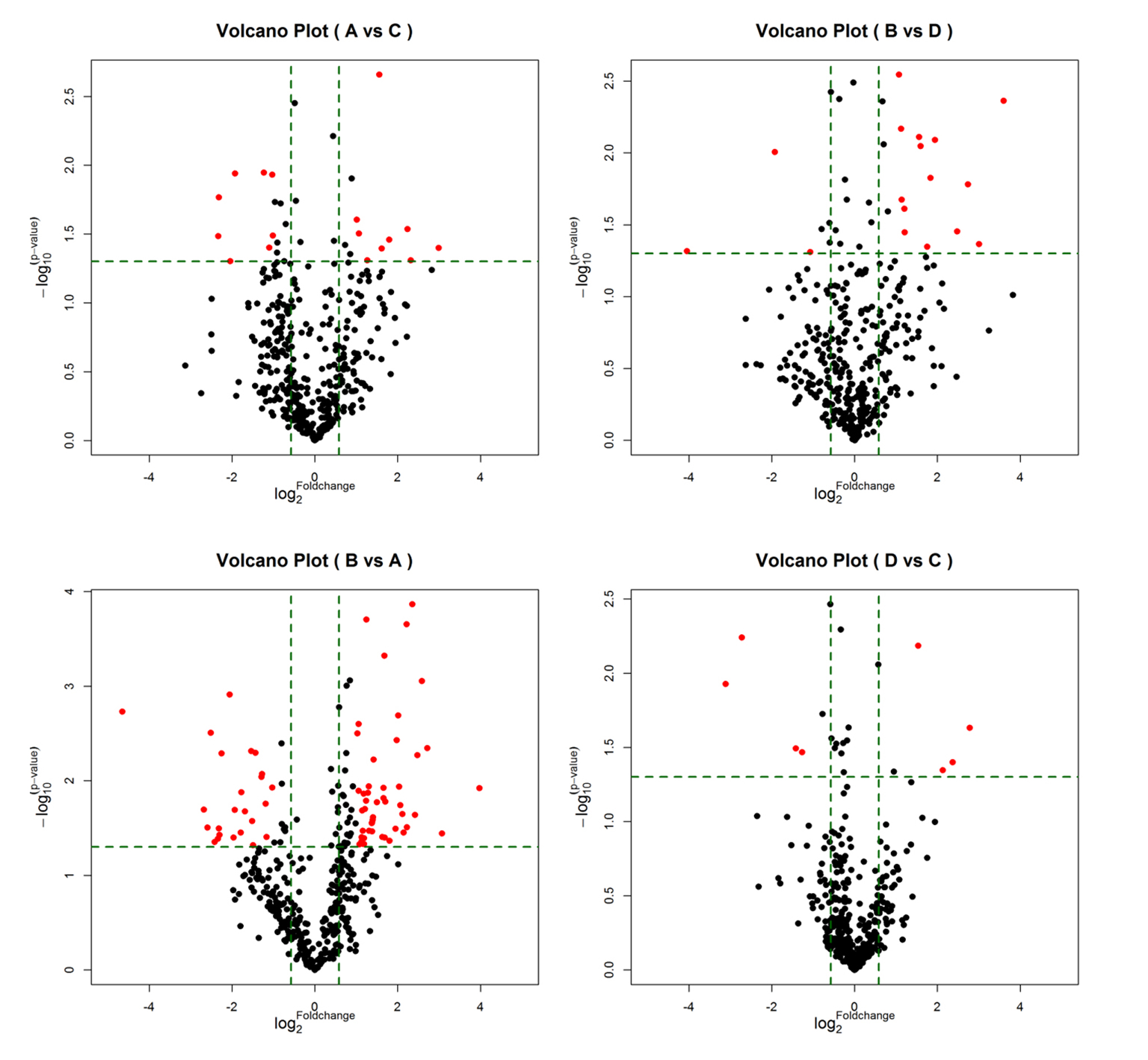

Supplement: Supplementary file 1 — Figure S1. Volcano plots of differential expression proteins. The vertical lines correspond to 2.0-fold up and down, respectively, and the horizontal line represents a p-value of 0.05. The red point in the plot represents the differentially protein with statistically significance. (A: gastric GIST with LGM; B: gastric GIST with HGM; C: corresponding adjacent normal tissues for LGM; D: corresponding adjacent normal tissues for HGM). (JPG 751 kb) [file 12876_2018_833_MOESM1_ESM.jpg]
